# Heat shock factor 5 correlated with immune infiltration serves as a prognostic biomarker in lung adenocarcinoma

**DOI:** 10.7150/ijms.51297

**Published:** 2021-01-01

**Authors:** Rusidanmu Aizemaiti, Zhigang Wu, Jie Tang, Haimeng Yan, Xiayi Lv

**Affiliations:** 1 Department of Thoracic Surgery, The First Affiliated Hospital of Zhejiang University, Qingchun Road 79, Hangzhou, China, 310009.; 2 Zhejiang University School of Medicine, Yuhangtang Road 866, Hangzhou, China, 310009.; 3 Bone Marrow Transplantation Center, The First Affiliated Hospital of Zhejiang University, Qingchun Road 79, Hangzhou, China, 310009.

**Keywords:** HSF5, lung adenocarcinoma, prognostic biomarker, immune infiltration, inflammatory activities

## Abstract

Lung adenocarcinoma (LUAD) is the predominant subtype of lung cancer with a relatively poor prognosis. The dramatic improvements of new immunotherapy strategies have shown promising results in lung cancer patients. This study aimed to elucidate the functions of immune-associated genes in LUAD prognosis and pathogenesis by analyzing public databases. We obtained expression profiles of LUAD patients from The Cancer Genome Atlas (TCGA) database and applied the ESTIMATE algorithm to calculate immune scores and stromal scores. A series of microenvironment-related genes with prognostic value was then identified. Of note, heat shock factor 5 (HSF5) was found to be decreased in LUAD patients and positively correlated with overall survival, which was further confirmed in the Gene Expression Omnibus (GEO) database. Moreover, Gene Ontology (GO) analysis based on the correlated genes of HSF5 demonstrated that HSF5 expression was significantly associated with the immune response and inflammatory activities. Based on the Tumor IMmune Estimation Resource (TIMER) and Gene Expression Profiling Interactive Analysis (GEPIA) datasets, HSF5 expression showed strong correlations with various immune cell infiltration and diverse immune marker sets. These findings suggest that HSF5 can be used as a promising biomarker for determining prognosis and immune infiltration in LUAD patients.

## Introduction

Lung cancer is the leading cause of cancer-related death worldwide, and lung adenocarcinoma (LUAD) represents the most prevalent subtype, which comprises approximately 40% of all lung cancer cases 1,2. Despite the achievements in understanding the pathogenesis of this disease and the development of multidisciplinary therapies, the clinical outcomes for LUAD patients remain poor, with an overall survival rate of less than 5 years 3,4. Therefore, there is an urgent need to discover specific prognostic factors for LUAD to predict the overall prognosis and improve the therapeutic management of patients.

Tumor cells are involved in extensive and dynamic crosstalk with the immune microenvironment, and this correlation plays crucial roles in cancer pathogenesis 5. Evading immune destruction is one of the hallmarks of cancer 6. The role of the immune system in lung oncogenesis is increasingly being investigated with a focus on the clinical responses to checkpoint blockade immunotherapies 7,8. Notably, the association between the expression levels of immune markers and the response to immune-based therapy has been explored in various studies 9,10. The immune-related markers show prognostic and predictive effects in lung cancer patients. For example, increased cytotoxic T cell lymphocytes (CTLs) appear to be associated with longer survival 11. The use of bioinformatic technologies based on expression profiling from public databases is an effective method to better understand the immune microenvironment in LUAD patients 12,13.

Heat shock factors (HSFs) are transcription factors that mediate responses to versatile forms of physiological and environmental stimuli 14. There are several HSF isoforms in the human genome, and studies have largely focused on HSF1 and HSF2 family members 14,15. HSF1 has been shown to play a vital role in innate immunity and immunosenescence 16. The deregulation of HSF activity is involved in various human diseases. For example, the compromised activation of HSF1 has been reported to be linked with the pathology of Huntington's disease and Parkinson's disease 17,18. Research has revealed that HSF1 drives carcinogenesis 19-21. However, HSF2 has appeared to decrease in a wide range of cancers and act as a tumor suppressor 22,23. To date, only a few studies have focused on HSF5, and its detailed functional characterization in tumors has not been performed 24.

In the present study, we evaluated the gene expression profiles of LUAD patients from The Cancer Genome Atlas (TCGA) database and identified a series of microenvironment-related genes with prognostic value. The positive correlation between HSF5 expression and overall survival was further validated in the Gene Expression Omnibus (GEO) database. Moreover, we explored the association of HSF5 with immune response and inflammatory activities, as well as immune cell infiltration and diverse immune marker sets in LUAD. This study revealed the crucial role of HSF5 in LUAD and an underlying mechanism between HSF5 and tumor-immune interactions.

## Materials and Methods

### Database

The RNA-Seq dataset of LUAD patients and corresponding clinical information were obtained from the TCGA database (https://gdc.nci.nih.gov/). We adopted two datasets (GSE31210 and GSE37745) from the GEO database. The data of GSE31210 were based on GPL570 platforms (HG-U133_Plus_2 Affymetrix Human Genome U133 Plus 2.0 Array) and included 226 lung adenocarcinoma patients. The GSE37745 data were based on GPL570 platforms and included 106 lung adenocarcinoma patients. Immune scores and stromal scores were calculated by the ESTIMATE algorithm of the downloaded database.

### DEG identification and functional enrichment analysis

All the LUAD patients were classified into high- and low-score groups based on their immune/stromal scores. Data analysis was performed by using the package edgeR. In this study, genes with a p value < 0.05 and | fold change | > 1.5 were defined as differentially expressed genes (DEGs). Database for Annotation, Visualization and Integrated Discovery (DAVID) (https://david-d.ncifcrf.gov/) was applied to analyze the gene functions.

### Survival analysis

Kaplan-Meier plots were constructed to investigate the correlation between gene expression and the overall survival of LUAD patients. The statistical significance of the correlation was tested by a log-rank test. The online Kaplan-Meier plotter database (http://kmplot.com/analysis/) was used to verify the prognostic values of the identified genes.

### Immune-associated analysis

The correlations between continuous variables were investigated by Spearman correlation analysis. Gene set variation analysis (GSVA) was conducted as previously described 25. Gene Ontology (GO) analysis of the most related genes was constructed by Heatmap. The GO gene set was obtained from the AmiGO 2 Web portal (http://amigo.geneontology.org/amigo/landing). Inflammatory-related metagenes were selected as described previously 26,27. The metagene expression values were determined by assessing the mean of the normalized expression values of all genes in a respective cluster 27. The Tumor IMmune Estimation Resource (TIMER) database (https://cistrome.shinyapps.io/timer/) was applied to estimate the abundance of immune infiltrates and the correlations between HSF5 expression and the gene markers of immune cells. The online database Gene Expression Profiling Interactive Analysis (GEPIA) (http://gepia.cancer-pku.cn/index.html) was used to further validate the significantly correlated genes in TIMER.

## Results

### Identification of DEGs based on immune scores and stromal scores

**Figure 1:**
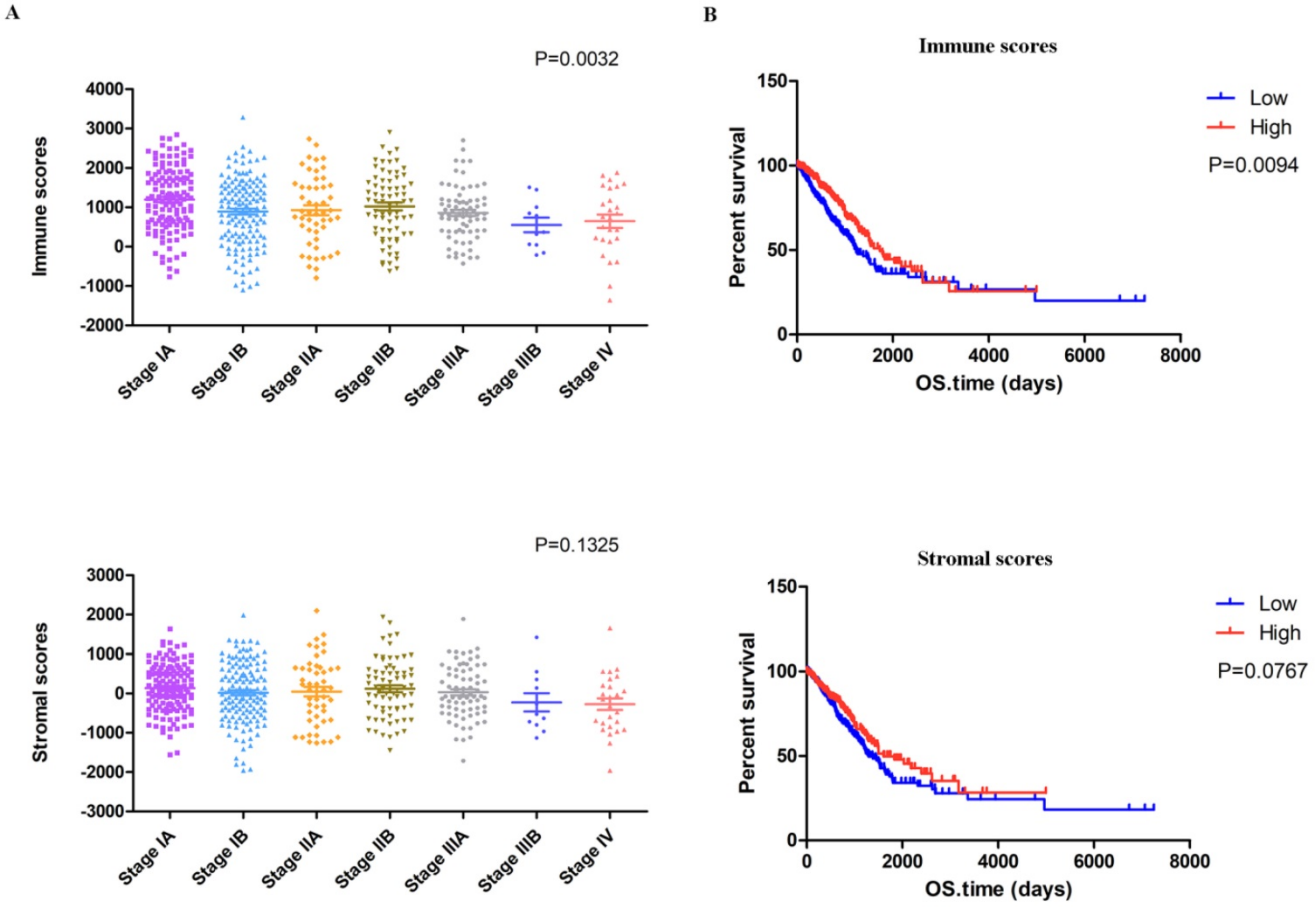

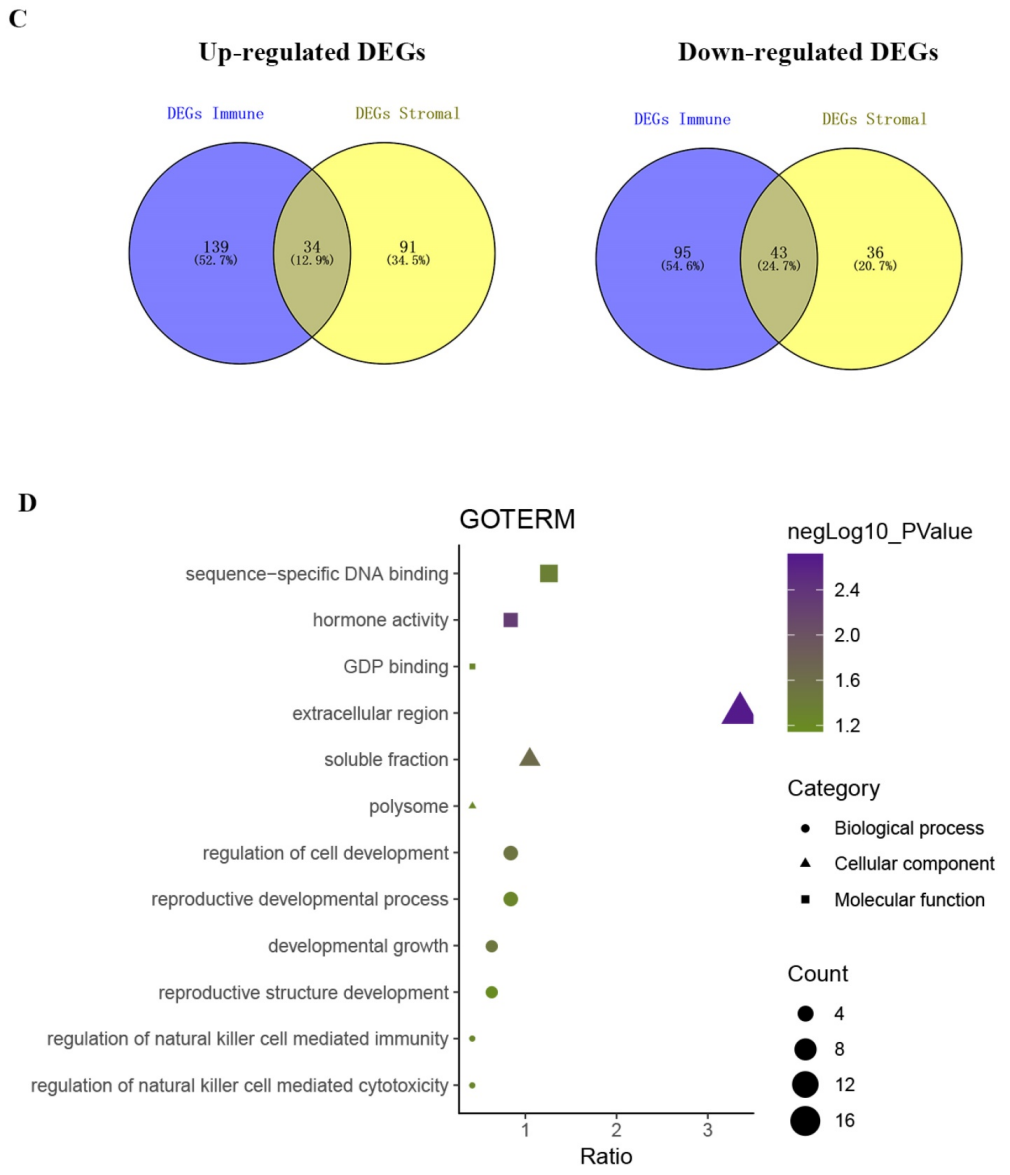
** Identification of DEGs based on immune scores and stromal scores.** (A) Distribution of immune scores and stromal scores for LUAD pathologic stage. (B) The Kaplan‐Meier survival curve reveals that high immune scores are associated with significantly longer overall survival. The high stromal score group showed a longer median overall survival than the low-score group, with no significant difference. (C) The commonly changed DEGs in the stromal and immune score groups (34 up- and 43 downregulated genes) were identified. (D) GO term enrichment analysis of the common DEGs.

The complete gene expression profiles and clinical information of 517 LUAD patients were downloaded from the TCGA database. We calculated the immune scores and stromal scores of all these patients with the ESTIMATE algorithm and plotted the distribution of the scores according to stage classifications of LUAD patients. As shown in Figure 1A, the immune scores were significantly associated with the pathologic stage, while the stromal scores displayed no statistically significant differences.

To explore the potential correlation of overall survival with immune scores and stromal scores, we classified the 517 LUAD cases into high- and low-score groups based on their scores (the top 259 scores are the high score group and the rest are the low score group). The Kaplan-Meier survival analysis (Figure 1B) demonstrated that the median overall survival of patients with high immune scores was longer than that of patients with low scores (1725 d vs. 1229 d, *p =* 0.0094). Moreover, patients in the high stromal score group had a longer median overall survival rate than patients in the low-score group, but with no significant difference (1600 d vs. 1293 d, *p* = 0.0767).

Setting *p <* 0.05 and | fold change | > 1.5 as the cut-off criteria, we identified 311 and 204 DEGs between the high and low immune score/stromal score groups, respectively. The integrated bioinformatic analysis revealed that 34 genes were commonly upregulated and 43 genes were commonly downregulated in the high-score group (Figure 1C). Subsequently, we conducted a functional enrichment analysis of the common DEGs with the DAVID gene annotation tool. As shown in Figure 1D, the top GO terms identified included extracellular region, soluble fraction, regulation of cell development and regulation of natural killer cell-mediated immunity.

### Survival analysis of the DEGs

**Figure 2:**
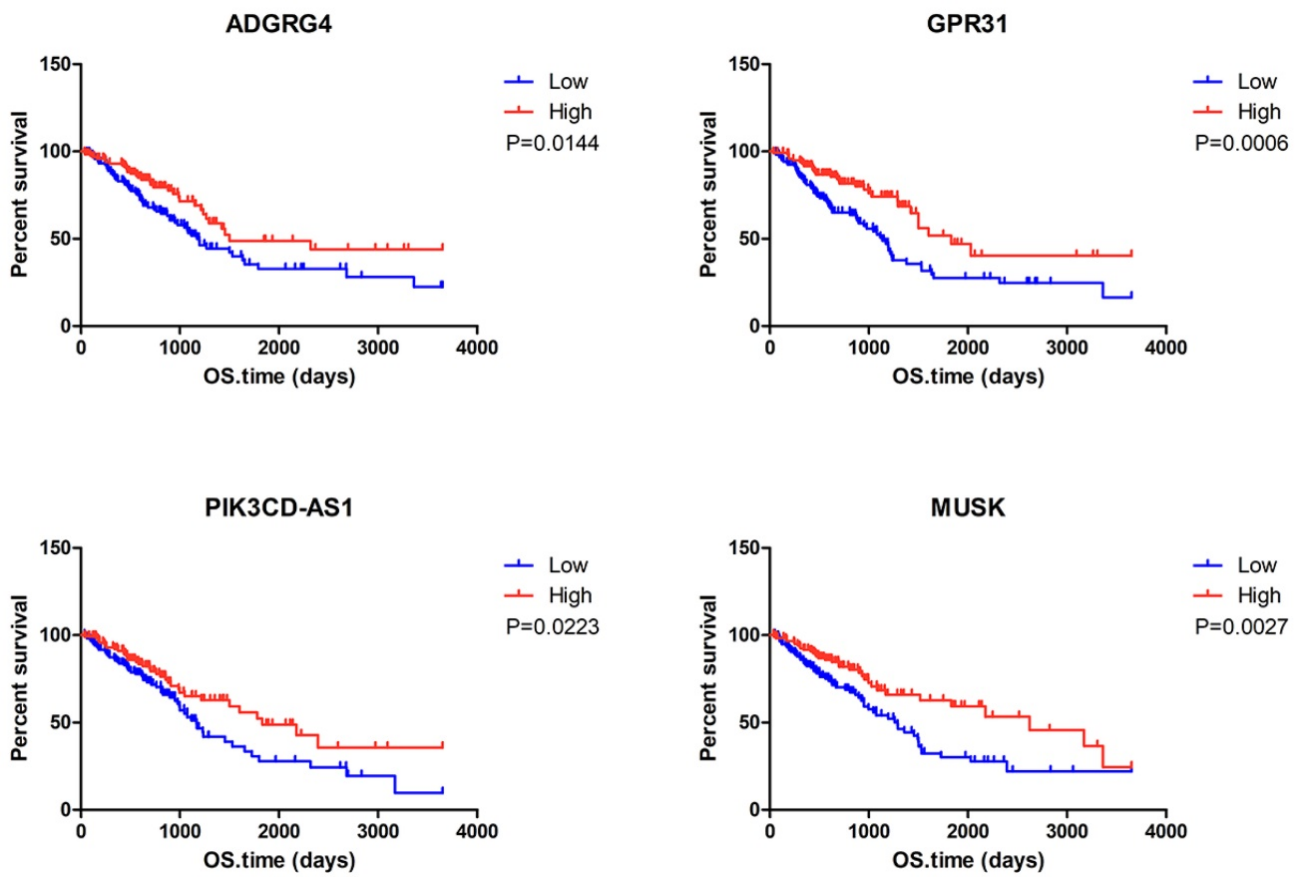

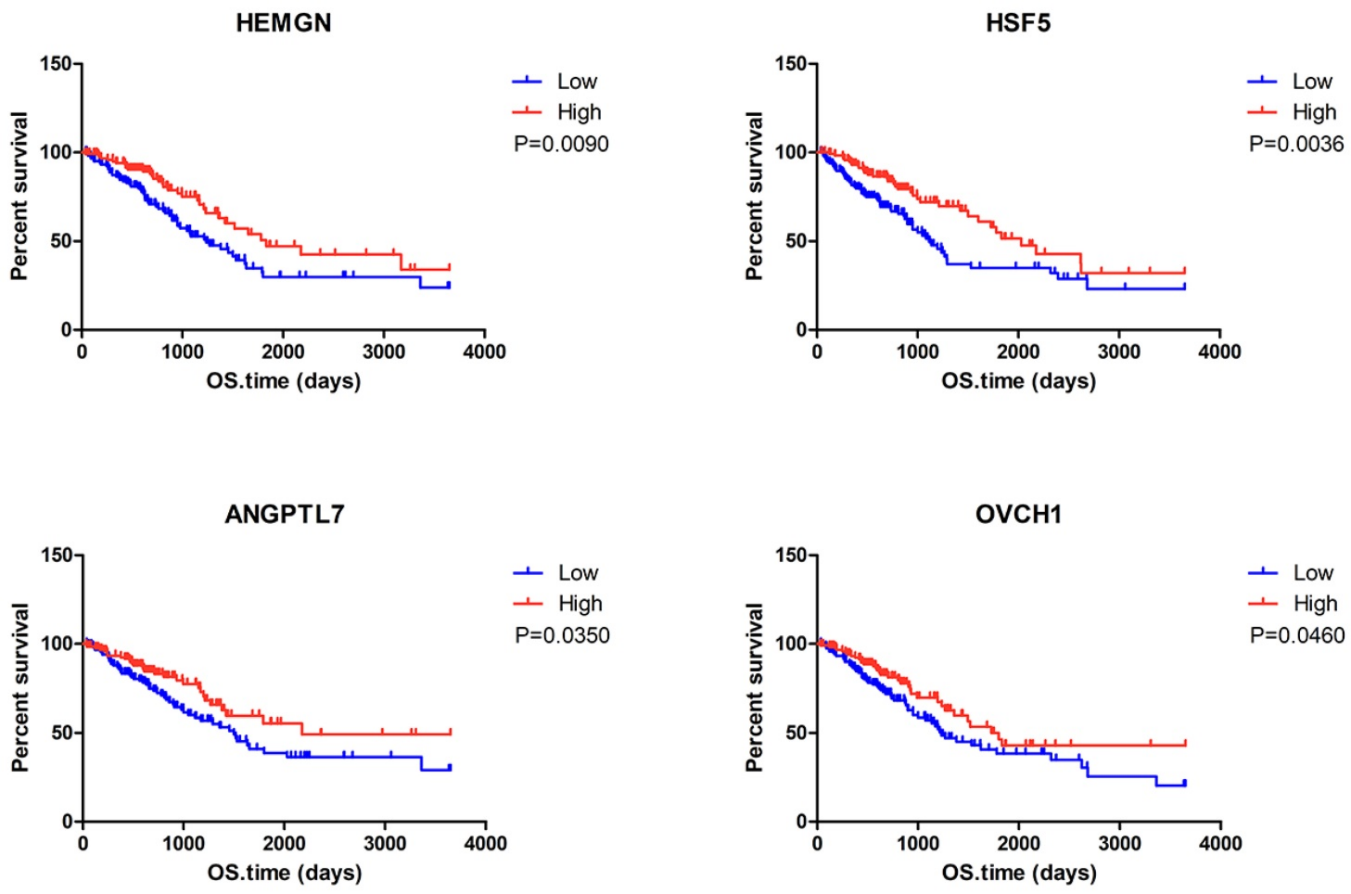
**Correlation between individual DEG expression and the overall survival of LUAD patients in TCGA.** Kaplan-Meier survival curves with the log-rank test are represented for the upregulated DEGs. ADGRG4, adhesion G protein-coupled receptor G4; GPR31,G protein-coupled receptor 31; HEMGN, hemogen; HSF5, heat shock transcription factor 5; PIK3CD-AS1, PIK3CD antisense RNA 1; MUSK, muscle associated receptor tyrosine kinase; ANGPTL7, angiopoietin like 7; OVCH1, ovochymase 1.

**Figure 3:**
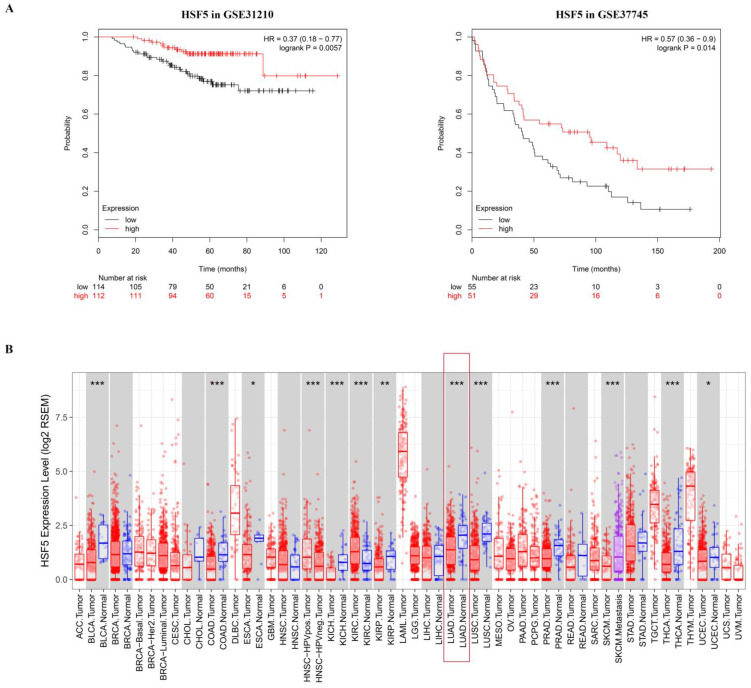
** The HSF5 prognostic value in GEO and expression in different cancers.** (A) HSF5 was further confirmed to be positively associated with the overall survival of LUAD patients in the GEO dataset (GSE31210 and GSE37745). (B) HSF5 expression levels in different types of human cancers were investigated by TIMER, red indicate the mRNA expression of HSF5 in tumor tissues; blue indicate the mRNA expression of HSF5 in normal tissues; purple indicate the mRNA expression of HSF5 in metastatic tissues (*p < 0.05, **p < 0.01, ***p < 0.001).

To determine the potential association of the total 77 DEGs with the overall survival of LUAD patients, we constructed Kaplan-Meier survival curves. Seventeen DEGs (8 upregulated DEGs and 9 downregulated DEGs) were significantly associated with overall survival in the log-rank test (*p* < 0.05). The 8 upregulated DEGs of prognostic value are shown in Figure 2. The prognostic evaluation of these genes in the Kaplan-Meier plotter database was consistent with our results (Supplementary Figure S1). Furthermore, we evaluated the prognostic potential of these genes in the GEO database (GSE31210 and GSE37745). Among the 8 upregulated DEGs, HSF5 was further confirmed to be positively associated with the overall survival of LUAD patients (Figure 3A). Additionally, we analyzed the differences in HSF5 expression in various tumor and normal tissues. As shown in Figure 3B, HSF5 expression was significantly lower in LUAD tissues than in adjacent normal tissues. Downregulated HSF5 expression was also observed in various cancers, including bladder urothelial carcinoma (BLCA), colon adenocarcinoma (COAD), kidney chromophobe (KICH), lung squamous cell carcinoma (LUSC), prostate adenocarcinoma (PRAD) and thyroid carcinoma (THCA). These results confirmed the decreased expression and prognostic value of HSF5 in LUAD.

### HSF5 is associated with the immune response and inflammatory activities in LUAD

**Figure 4:**
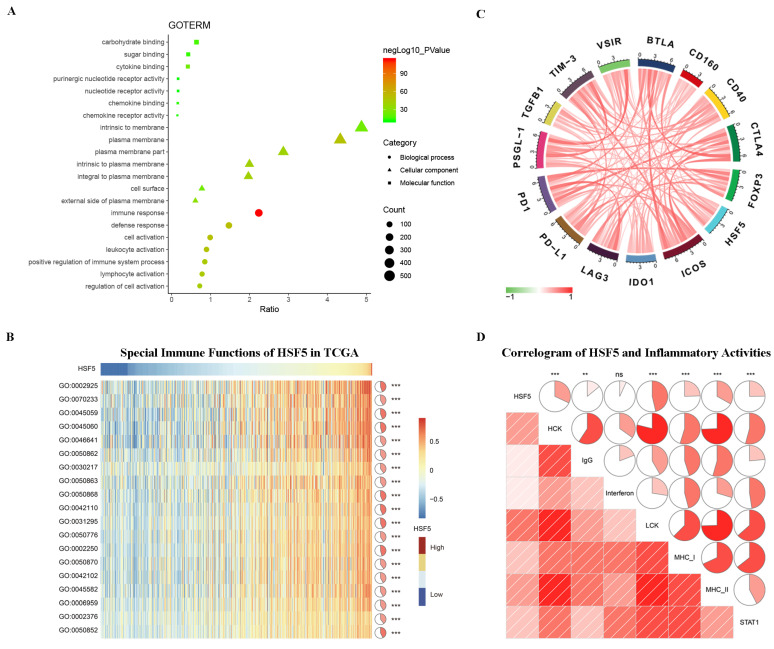
** HSF5-related immune response and inflammatory activities in LUAD.** (A) The biological functions of the HSF5-related genes were investigated by GO term enrichment analysis. (B) The correlation between HSF5 and the immune response in the TCGA dataset. (C) The relationship between HSF5 and immune checkpoint members. (D) The correlation between HSF5 and inflammatory activities. ns, **, and *** indicate no significant difference, *p*<0.01, and *p*<0.001, respectively. GO: 0002925, positive regulation of humoral immune response mediated by circulating immunoglobulin. GO: 0070233, negative regulation of T cell apoptotic process. GO: 0045059, positive thymic T cell selection. GO: 0045060, negative thymic T cell selection. GO: 0046641, positive regulation of alpha-beta T cell proliferation. GO: 0050862, positive regulation of T cell receptor signaling pathway. GO: 0030217, T cell differentiation. GO: 0050863, regulation of T cell activation. GO: 0050868, negative regulation of T cell activation. GO: 0042110, T cell activation. GO: 0031295, T cell costimulation. GO: 0050776, regulation of immune response. GO: 0002250, adaptive immune response. GO: 0050870, positive regulation of T cell activation. GO: 0042102, positive regulation of T cell proliferation. GO: 0045582, positive regulation of T cell differentiation. GO: 0006959, humoral immune response. GO: 0002376, immune system process. GO: 0050852, T cell receptor signaling pathway.

According to the above results, HSF5 may play a crucial role in the biological functions of LUAD, which has not been reported previously. To better understand the relevance and underlying mechanisms of HSF5 in LUAD, 1296 genes were screened to be significantly correlated with HSF5 based on the TCGA dataset and Spearman's correlation analysis (Spearman R > 0.3). The biological functions of these related genes were further analyzed by DAVID. GO analysis revealed that the related genes were mainly involved in the immune response, lymphocyte activation, the inflammatory response and the regulation of T cell activation (Figure 4A).

Subsequently, we performed GSVA analysis to explore the relationship between HSF5 and the immune response in LUAD (Figure 4B). The results showed that HSF5 was positively correlated with the adaptive immune response, T cell costimulation, T cell activation, the T cell receptor signaling pathway, the humoral immune response and the regulation of immune response. These results indicated that HSF5 may play an important role in the immune response, especially in T cell immunity. Additionally, we conducted a Spearman's correlation analysis on the expression of HSF5 and a variety of immune checkpoints from the TCGA dataset, such as PD-L1, PD1, CTLA-4, and IDO1. As shown in Figure 4C, HSF5 demonstrated a high correlation with ICOS and BTLA, followed by PSGL-1, CTLA4 and TIM-3.

To further understand HSF5-related inflammatory activities, we analyzed seven metagenes by a previously described method 26,27. We found that HSF5 expression was positively associated with HCK, LCK, MHC-I, MHC-II, STAT1, and IgG but not significantly associated with interferon in the TCGA database (Figure 4D). This result revealed that HSF5 was mainly associated with the activities of macrophages, the signal transduction of T cells, B cells and antigen-presenting cells. Together, these findings indicate that HSF5 has crucial immune and inflammatory functions in LUAD.

### HSF5 expression is correlated with the immune infiltration level in LUAD

**Figure 5:**
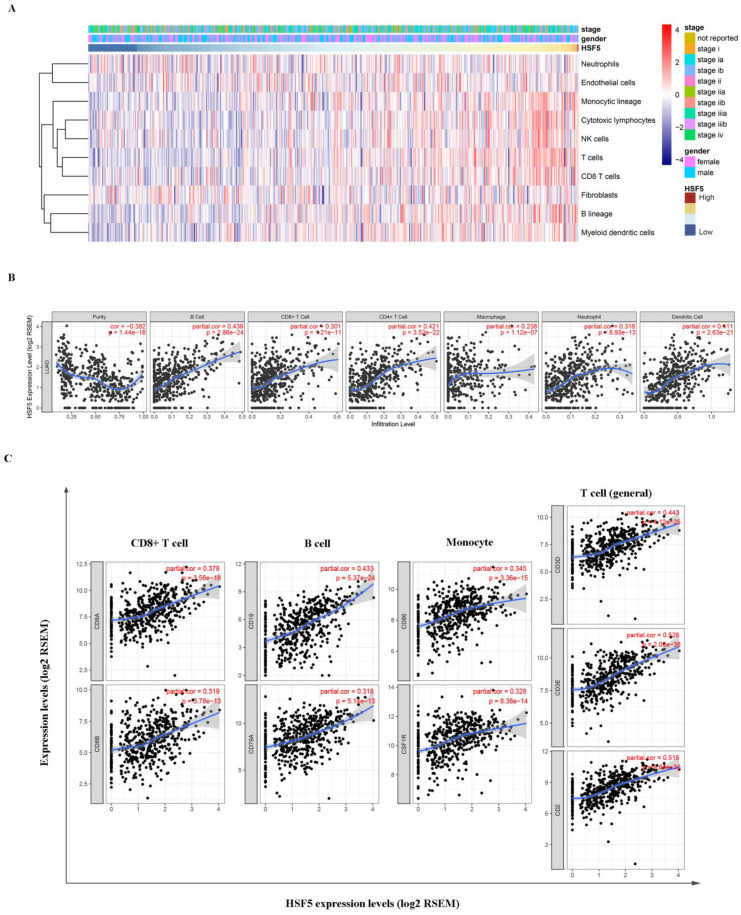
** HSF5 expression is correlated with the immune infiltration level in LUAD.** (A) The association between HSF5 expression and immune cell populations was evaluated by the Microenvironment Cell Populations-counter method. (B) The strong positive relevance between HSF5 and infiltrating levels of B cells, CD8+ T cells, CD4+ T cells, macrophages, neutrophils and dendritic cells were confirmed in the TIMER database. (C) The correlation of HSF5 and immune marker sets of various immune cells in LUAD based on TIMER database (CD8A and CD8B of CD8+T cells, CD86 and CD115 of monocytes, CD19 and CD79A of B cells, CD3D, CD3E and CD2 of general T cells).

Based on the TCGA dataset, we assessed the relationship between HSF5 expression and various immune cell populations by the Microenvironment Cell Populations-counter method as previously described 28. As shown in Figure 5A, HSF5 was significantly associated with T cells, B lineage, monocytic lineage and cytotoxic lymphocytes. In addition, we evaluated the correlations of HSF5 with immune infiltration levels in LUAD from TIMER. The result showed that the HSF5 expression level has strong positive relevance with infiltrating levels of B cells (*r =* 0.439, *P =*2.86e-24), CD8+ T cells (*r =* 0.301, *P =*1.21e-11), CD4+ T cells (r= 0.421, *P =* 3.52e-22), macrophages (*r =* 0.238, *P =* 1.12e-07), neutrophils (*r =* 0.318, *P =* 8.93e-13) and dendritic cells (DCs) (*r =*0.411, *P =* 2.63e-21) in LUAD (Figure 5B). These findings suggest that HSF5 plays a specific role in immune infiltration in LUAD, especially in T cells and B cells.

### Correlation of HSF5 expression and immune marker sets

To validate the relationship between HSF5 and immune cells, we further estimated the correlation between HSF5 and the immune marker genes of various immune cells in LUAD based on the TIMER and GEPIA databases. We focused on the association between HSF5 and immune marker sets of diverse immune cells, including CD8+ T cells, T cells (general), B cells, monocytes, tumor-associated macrophages (TAMs), M1 and M2 macrophages, neutrophils, natural killer (NK) cells and DCs (Table 1). Specifically, we showed that CD8A and CD8B of CD8+ T cells, CD3D, CD3E, and CD2 of general T cells, CD86 and CD115 of monocytes, and CD19 and CD79A of B cells are significantly associated with HSF5 expression (Figure 5C). Subsequently, we employed the GEPIA dataset to validate the above correlations (Table 2, Supplementary Figure S2). These findings were consistent with the correlation analysis between HSF5 expression and immune cells, indicating that HSF5 plays a vital role in the immune response in the microenvironment of LUAD.

## Discussion

**Figure 6:**
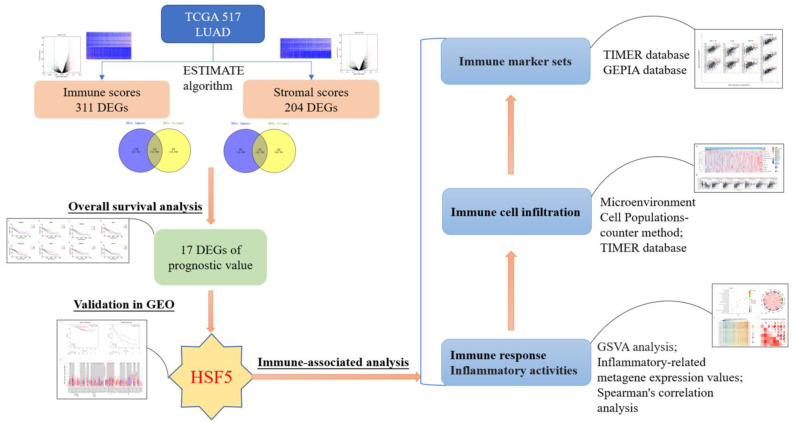
** Work flow of the current study.** The expression profiles of LUAD patients from TCGA database and immune scores and stromal scores, calculated from the ESTIMATE algorithm. HSF5 (microenvironment-related genes with prognostic value) was then identified, and further confirmed in the GEO database. Moreover, GO and GSVA analyses demonstrated that HSF5 expression was significantly associated with the immune response and inflammatory activities. According to TIMER and GEPIA datasets, the HSF5 expression significantly correlated with various immune cell infiltration and diverse immune marker sets.

LUAD remains one of the most aggressive and fatal tumor types despite the dramatic improvements of new therapeutic strategies 3,4. In this study, we analyzed microenvironment-associated genes of prognostic value to LUAD based on the TCGA and GEO databases. HSF5 was found to be decreased in LUAD patients and positively correlated with overall survival. Furthermore, we demonstrated that HSF5 is significantly associated with immune response and inflammatory activities, as well as immune cell infiltration and diverse immune marker sets (Figure 6).

The ESTIMATE algorithm is designed to calculate immune and stromal scores according to gene expression data and signatures 29. Various studies have employed this algorithm to explore the microenvironment of prostate cancer 30, colon cancer 31 and glioblastoma 32. Here, we first assessed the infiltration of stromal and immune cells in LUAD patients based on the ESTIMATE algorithm. The immune scores were significantly correlated to the pathologic stage and overall survival of LUAD patients. Consistent with our results, a recent study revealed that a high immune score was associated with better progression-free survival (PFS) of lung cancer patients based on clinical data 33. The immune microenvironment is widely recognized to influence lung cancer outcomes by contributing to inflammation, angiogenesis, immune modulation and the response to therapies 34,35. Much effort has been put into exploring immune biology and developing effective immunotherapeutic strategies for lung cancer 36,37. Thus, integrating and reanalyzing genomic profiles from public databases are important to obtain a better understanding of the immune microenvironment in LUAD.

The common DEGs between the high and low immune score/stromal score groups were identified, followed by an overall survival analysis. The results demonstrated that 17 DEGs were significantly associated with the prognosis of LUAD patients. The prognostic value of these genes was also confirmed in the Kaplan-Meier plotter database. Of note, HSF5 was further verified to be positively associated with the overall survival of LUAD patients from the GEO database. Additionally, we revealed that HSF5 expression was significantly downregulated in LUAD tissues compared with adjacent normal tissues. Moreover, lower HSF5 expression was also observed in bladder, colon, kidney, prostate and thyroid cancers based on TIMER. Consistent with this result, another HSF family member, HSF2, has been reported to be frequently decreased in several human malignancies and acts as a tumor suppressor 23. However, the major stress-responsive factor HSF1 appears to support cancer cell growth, survival and metastasis 19,21. HSF5 has not previously been connected to cancer, and in this study, we demonstrated the low expression and prognostic value of HSF5 in LUAD.

HSF5 belongs to the heat shock transcription factor family, which is involved in differentiation, reproduction, and stress-induced adaptation 14. Previous studies have revealed that HSF5 plays a critical role in germ cell development and meiotic progression 24,38. However, the functional characterization of HSF5 involved in cancer and the immune response has not been conducted. In this study, we showed that HSF5-related genes were mainly enriched in the immune response, lymphocyte activation, and the inflammatory response in LUAD. Further GSVA analysis also demonstrated that HSF5 was positively correlated with the adaptive immune response, T cell activation, the T cell receptor signaling pathway, and the regulation of the immune response. Consistently, another HSF family member, HSF1, has been reported to enable the normal function of the immune system 16,39. These results indicated the potential role of HSF5 in the immune response, especially in T cell immunity.

Another important aspect of this study is that HSF5 expression is associated with diverse immune cell infiltration and immune marker sets in LUAD. Our analysis demonstrated that there was a strong positive correlation between HSF5 expression level and infiltrating levels of B cells, CD8+ T cells, CD4+ T cells, macrophages, neutrophils and DCs in LUAD. Importantly, the correlation between HSF5 expression and the marker genes of these immune cells implicates the function of HSF5 in regulating tumor immunology. In lung cancer, the prognostic and predictive significance of immune markers has been elucidated in various studies 8,40. CD8+ T cells, CD4+ T cells and mature DCs appeared to be associated with good prognosis. Regulatory T cells (Tregs), immature DCs, and M2 macrophages were shown to be related to poor outcomes 41. In recent years, immunotherapy has changed the therapeutic strategy and shown promising results in lung cancer patients 7,42. Ongoing immunotherapy biomarker research is essential to develop more accurately customized immunotherapy strategies 8. Our findings suggest that HSF5 plays an important role in the regulation of immune infiltration and may be a biomarker for immunotherapy in LUAD. The detailed function and underlying mechanism of HSF5 need to be further investigated.

In conclusion, we explored the microenvironment-associated genes of prognostic value to LUAD through integrated bioinformatics analysis. We found that HSF5 was downregulated and positively correlated with the overall survival of LUAD patients.

Moreover, HSF5 is involved in the immune response and potentially contributes to the regulation of B cells, CD8+ T cells, CD4+ T cells and DCs. These findings suggest that HSF5 plays a crucial role in the immune microenvironment and as a prognostic biomarker in LUAD patients.

## Supplementary Material

Supplementary figures.

## Figures and Tables

**Table 1:** Correlation analysis between HSF5 and relate genes and markers of immune cells in TIMER

Description	Gene markers	LUAD					
		None		Purity		Age	
		Cor	*P*	Cor	*P*	Cor	*P*
CD8+ T cell	CD8A	0.475	***	0.378	***	0.466	***
	CD8B	0.401	***	0.319	***	0.399	***
T cell (general)	CD3D	0.547	***	0.443	***	0.534	***
	CD3E	0.612	***	0.526	***	0.6	***
	CD2	0.608	***	0.515	***	0.596	***
B cell	CD19	0.517	***	0.433	***	0.525	***
	CD79A	0.425	***	0.318	***	0.428	***
Monocyte	CD86	0.463	***	0.345	***	0.46	***
	CD115 (CSF1R)	0.437	***	0.328	***	0.433	***
TAM	CCL2	0.297	***	0.197	***	0.288	***
	CD68	0.353	***	0.247	***	0.356	***
	IL10	0.403	***	0.299	***	0.404	***
M1 Macrophage	INOS (NOS2)	0.056	0.206	-0.024	0.596	0.041	0.370
	IRF5	0.33	***	0.227	***	0.337	***
	COX2 (PTGS2)	-0.088	0.0471	-0.083	0.0645	-0.087	0.057
M2 Macrophage	CD163	0.351	***	0.243	***	0.354	***
	VSIG4	0.349	***	0.253	***	0.348	***
	MS4A4A	0.414	***	0.306	***	0.414	***
Neutrophils	CD66b (CEACAM8)	0.241	***	0.228	***	0.217	***
	CD11b (ITGAM)	0.418	***	0.31	***	0.418	***
	CCR7	0.63	***	0.561	***	0.623	***
Natural killer cell	KIR2DL1	0.134	*	0.074	0.1	0.128	*
	KIR2DL3	0.139	*	0.043	0.337	0.165	**
	KIR2DL4	0.111	0.0121	0.027	0.555	0.119	*
	KIR3DL1	0.122	*	0.059	0.189	0.121	*
	KIR3DL2	0.22	***	0.144	*	0.223	***
	KIR3DL3	0.024	0.594	-0.003	0.946	0.019	0.676
	KIR2DS4	0.144	*	0.058	0.201	0.154	**
Dendritic cell	HLA-DPB1	0.504	***	0.413	***	0.487	***
	HLA-DQB1	0.357	***	0.254	***	0.341	***
	HLA-DRA	0.474	***	0.376	***	0.46	***
	HLA-DPA1	0.471	***	0.379	***	0.457	***
	BDCA-1 (CD1C)	0.435	***	0.369	***	0.412	***
	BDCA-4 (NRP1)	0.101	0.0216	0.05	0.266	0.09	0.0484
	CD11c (ITGAX)	0.451	***	0.338	***	0.463	***
Th1	T-bet (TBX21)	0.477	***	0.378	***	0.465	***
	STAT4	0.476	***	0.371	***	0.463	***
	STAT1	0.269	***	0.161	***	0.272	***
	IFN-γ (IFNG)	0.326	***	0.22	***	0.324	***
	TNF-α (TNF)	0.306	***	0.178	***	0.291	***
Th2	GATA3	0.388	***	0.266	***	0.38	***
	STAT6	0.09	0.0416	0.104	0.0204	0.072	0.117
	STAT5A	0.489	***	0.38	***	0.476	***
	IL13	0.244	***	0.178	***	0.225	***
Tfh	BCL6	0.019	0.671	0.007	0.872	0.003	0.504
	IL21	0.269	***	0.206	***	0.289	***
Th17	STAT3	0.01	0.826	0.03	0.511	-0.001	0.986
	IL17A	0.262	***	0.199	***	0.274	***
Treg	FOXP3	0.505	***	0.391	***	0.501	***
	CCR8	0.536	***	0.429	***	0.538	***
	STAT5B	0.302	***	0.295	***	0.303	***
	TGFβ (TGFB1)	0.295	***	0.2	***	0.28	***
T cell exhaustion	PD-1 (PDCD1)	0.473	***	0.38	***	0.466	***
	CTLA4	0.54	***	0.429	***	0.542	***
	LAG3	0.363	***	0.264	***	0.369	***
	TIM-3 (HAVCR2)	0.448	***	0.329	***	0.443	***
	GZMB	0.247	***	0.119	*	0.249	***

LUAD, lung adenocarcinoma; TAM, tumor-associated macrophage; Th, T helper cell; Tfh, Follicular helper T cell; Treg, regulatory T cell; Cor, R value of Spearman's correlation; None, correlation without adjustment. Purity, correlation adjusted by purity. Age, correlation adjusted by age. *P < 0.01; **P < 0.001; ***P < 0.0001.

**Table 2:** Correlation analysis between HSF5 and relate genes and markers of immune cells in GEPIA

Description	Gene markers	LUAD			
		Tumor		Normal	
		R	*P*	R	*P*
CD8+ T cell	CD8A	0.45	***	-0.035	0.79
	CD8B	0.37	***	0.07	0.6
	CD8A and CD8B	0.42	***	0.014	0.01
T cell (general)	CD3D	0.51	***	-0.071	0.59
	CD3E	0.61	***	0.12	0.38
	CD2	0.6	***	-0.09	0.5
	CD3D, CD3E and CD2	0.89	***	-0.04	0.76
B cell	CD19	0.49	***	0.16	0.22
	CD79A	0.4	***	0.011	0.94
	CD19 and CD79A	0.45	***	0.039	0.77
Monocyte	CD86	0.48	***	0.043	0.75
	CD115 (CSF1R)	0.46	***	0.21	0.1
	CD86 and CD115(CSF1R)	0.48	***	0.17	0.19

LUAD, lung adenocarcinoma; tumor, correlation analysis in tumor tissue of TCGA; normal, correlation analysis in normal tissue of TCGA. ***P < 0.0001.
