# Supplementary material for: Heat shock factor 5 correlated with immune infiltration serves as a prognostic biomarker in lung adenocarcinoma
Source: Int J Med Sci. 2021 Jan 1;18(2):448–58. doi: 10.7150/ijms.51297 (PMC7757139; doi:10.7150/ijms.51297)
Supplement: Supplementary file 1 — Supplementary figures. [file ijmsv18p0448s1.pdf]

Supplementary Figures

Supplementary figure 1.

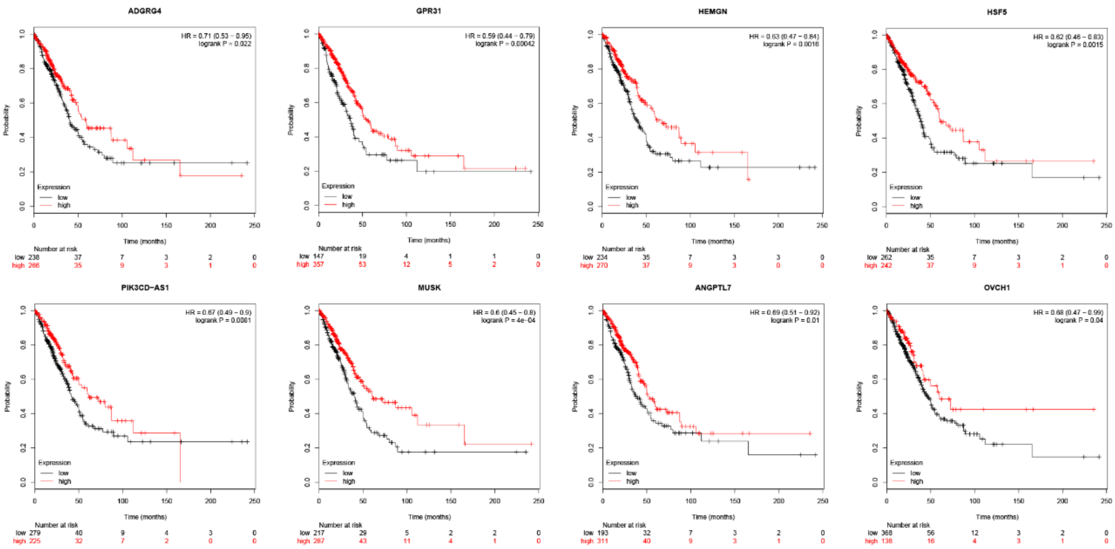

Supplementary figure 1. Verification of the genes of prognostic value in the Kaplan-Meier plotter database.

Supplementary Figure 2.

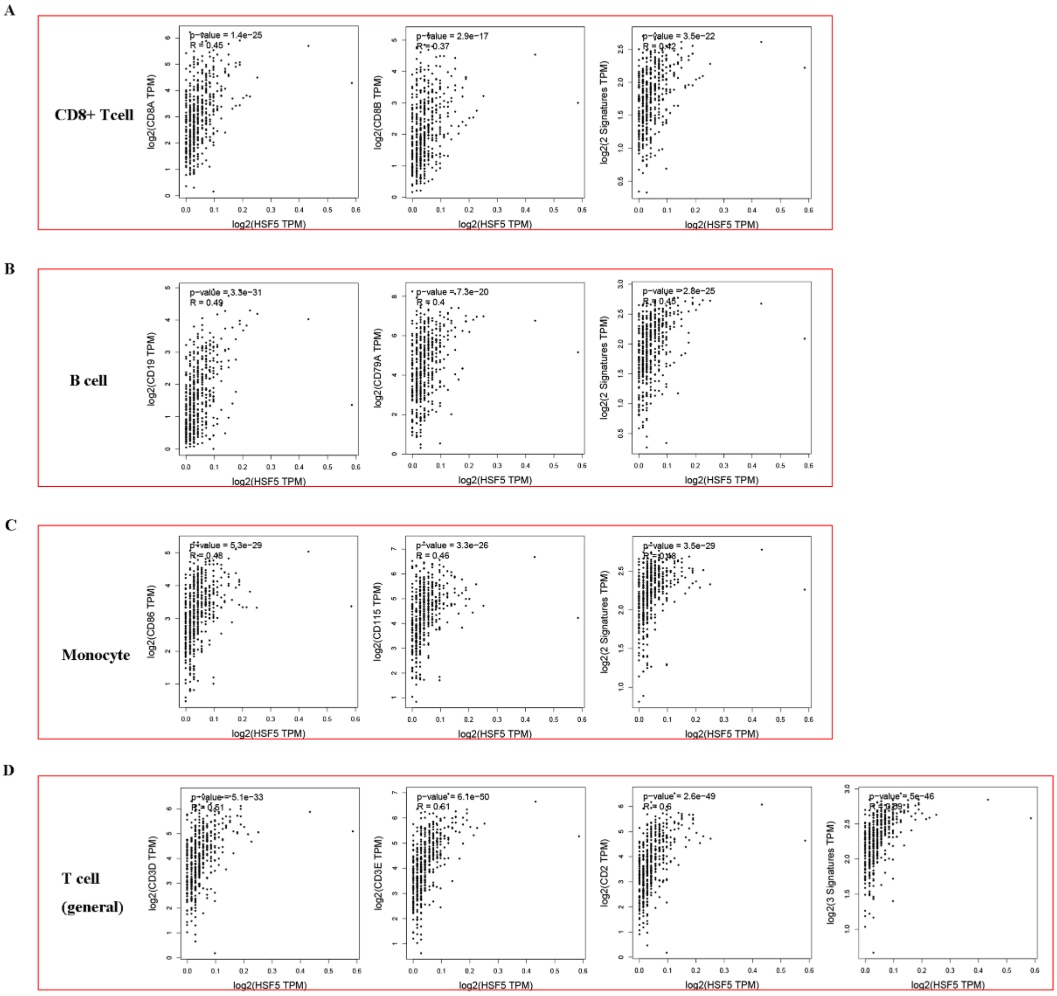

Supplementary Figure 2. Correlation of HSF5 and immune marker sets of various immune cells in LUAD. CD8A and CD8B of CD8+T cells (A), CD86 and CD115 of monocytes (B), CD19 and CD79A of B cells (C), CD3D, CD3E and CD2 of general T cells (D), are significantly associated with HSF5 expression based on GEPIA database.
